# Efficacy of different spinal orthoses for pain management, functional improvement, and quality of life in osteoporotic vertebral fractures: a mini review

**DOI:** 10.3389/fresc.2024.1506279

**Published:** 2024-12-23

**Authors:** M. B. Borg, M. Battaglia, M. Invernizzi, A. Baricich

**Affiliations:** ^1^ Department of Health Sciences, Università del Piemonte Orientale “Amedeo Avogadro”, Novara, Italy; ^2^ Physical Medicine and Rehabilitation Unit, AOU Maggiore Della Carità University Hospital, Novara, Italy; ^3^ Translational Medicine, Dipartimento Attività Integrate Ricerca e Innovazione (DAIRI), Azienda Ospedaliera Santi Antonio e Biagio e Cesare Arrigo, Alessandria, Italy; ^4^ Department of Biomedical Sciences, Humanitas University, Milan, Italy; ^5^ Rehabilitation Unit, IRCSS Humanitas Research Hospital, Milan, Italy

**Keywords:** osteoporotic vertebral fractures (OVFs), spinal orthoses, pain management, functional improvement, quality of life

## Abstract

Osteoporotic vertebral fractures (OVFs) significantly impair the quality of life in older adults. Spinal orthoses are commonly used, but their effectiveness is debated. Thus, we aimed to evaluate the impact of different spinal orthoses on pain, functionality, and quality of life in patients with OVFs. A review of PubMed, Scopus, PEDro, and Cochrane Library was conducted, covering studies from 2000 to 2024. Studies assessing spinal orthoses’ effects on pain, functionality, and quality of life in adults with OVFs were included. Ten studies involving 970 participants were included. Several studies reported significant improvements in pain, functionality, and quality of life with spinal orthoses. Dynamic hyperextension orthoses, such as Spinomed® and Spinfast®, showed potential benefits, particularly in enhancing trunk muscle strength. In conclusion, spinal orthoses may help manage pain and improve functionality and quality of life in OVFs. However, further high-quality trials are needed to confirm their efficacy.

## 1 Introduction

Osteoporotic vertebral fractures (OVFs) are a common and debilitating consequence of osteoporosis, a systemic skeletal disease characterized by low bone mass and microarchitectural deterioration of bone tissue (1).

OVFs are associated with substantial morbidity and mortality, significantly impacting patients' quality of life (2). The pathophysiology of OVFs involves a complex interplay of factors, including decreased bone density, altered bone microarchitecture, and increased bone fragility (3). Clinically, OVFs often present with acute back pain, progressive deformity, loss of height, and disability. Current treatment strategies for OVFs aim to manage pain, prevent further fractures, and improve function and quality of life. These approaches include pharmacological therapies, surgical treatments, physical therapies, lifestyle modifications, and the use of orthotic devices (4, 5). Among these, spinal orthoses have gained particular attention for their potential to provide external support, reduce pain, and enhance functional status in patients with stable fractures and no neurological impairments (6).

There are various types of spinal orthoses, which can be either custom-made or prefabricated. They can be categorized based on the area of the spine they support, or on their specific properties. In this mini-review, the following types of spinal orthoses for osteoporotic fragility vertebral fractures were considered (7):

1. Rigid Orthosis: rigid orthoses are spinal braces made from hard materials such as plastic or metal, designed to provide maximal support and immobilization to the spine. These devices restrict movement in multiple planes, reducing the load on the vertebral bodies and promoting spinal alignment. They are typically prescribed for acute traumatic fractures or post-surgical stabilization. They provide strong immobilization and protection against further injury. However, they could also lead to discomfort, potential muscle atrophy due to limited movement, and challenges in adherence to long-term use (8).
2. Soft Orthosis: soft orthoses, also known as flexible braces, are constructed from elastic or foam materials, offering support with greater flexibility compared to rigid orthoses. They allow for some spinal movement while providing moderate support and compression. These are often used for less severe fractures or for patients requiring prolonged use, where comfort is a priority. Advantages include greater comfort and ease of use, less impacting muscle function. However, disadvantages include reduced support and immobilization, which may be insufficient for more severe fractures.
3. Dynamic Orthosis: dynamic orthoses, such as the Spinfast® and the Spinomed® braces, are designed to encourage muscle activation and posture correction through biofeedback mechanisms. These braces are more flexible and adjust to the patient's movement, promoting active engagement of the back muscles during daily activities. They are often indicated for chronic management, focusing on improving functionality and reducing pain over time. Advantages include the potential for improving muscle strength and posture, with less discomfort than rigid braces. Disadvantages include a potentially lower degree of stabilization compared to rigid orthoses, and the need for patient compliance and correct usage to achieve therapeutic benefits.

Spinal orthoses, including rigid, soft, or dynamic braces, are commonly employed to manage of OVFs. However, the efficacy of these devices remains a topic of ongoing debate, with studies reporting mixed results (6, 9, 10). Some evidence suggests that dynamic orthoses may offer significant benefits for patients with sub-acute OVFs. Yet, very low-quality evidence indicates no significant differences in outcomes when comparing dynamic, rigid, and soft braces in the management of acute fractures (11). Consequently, a recent systematic review concluded that, due to the variability in study quality and the limitations of previous research, a general recommendation for the use of spinal orthoses in treating osteoporotic vertebral fractures is not feasible, as no clear evidence supports the superiority of any specific orthosis (12).

In summary, despite the widespread use of spinal orthoses for osteoporotic vertebral fractures (OVFs), there is no clear consensus on their effectiveness in key outcomes such as pain relief, functional improvement, and quality of life. Previous reviews have explored these devices, but a thorough evaluation comparing different types of orthoses is still missing. This mini-review addresses this gap by assessing the efficacy of various orthoses in managing pain, enhancing functionality, and improving quality of life in OVF patients, aiming to identify any orthosis that demonstrates superior efficacy and to inform clinical practice and future research efforts.

## 2 Methods and findings

### 2.1 Search and selection strategy

Two investigators independently performed a systematic search in PubMed/MEDLINE, Scopus, PEDro, and the Cochrane Library (PROSPERO registration number CRD42024573116), focusing on studies published from 2000 onwards in English and involving human participants. This time frame was chosen to prioritize research on contemporary spinal orthoses, as technological advancements render older models less relevant. The search employed Medical Subject Headings (MeSH) and keywords based on the Population, Intervention, Comparator, and Outcome (PICO) framework. The eligibility criteria were as follows: adults aged 18 years or older with osteoporotic vertebral fractures (Population), studies investigating the use of spinal orthoses (Intervention), studies comparing different types of spinal orthoses, comparing a spinal orthosis to no intervention, or studies with no comparator (Comparator), and outcomes related to pain, functionality (e.g., back extensor strength and gait), and quality of life (Outcomes). Experimental studies, including randomized controlled trials (RCTs), cohort studies, case-control studies, and prospective studies, were included, regardless of the presence of a control group. Non-experimental designs, observational studies, reviews, and studies not reporting the relevant outcomes were excluded. After removing duplicates, the investigators independently reviewed titles and abstracts, resolving discrepancies through discussion or, if needed, consultation with a third reviewer. Full texts of the selected studies were then evaluated, and reference lists were scanned for additional relevant studies. The final search was completed on July 25th, 2024, with any remaining disagreements settled by a third author.

The search string used was: [(osteoporosis OR osteoporotic OR fragility) AND fracture] AND (brace OR bracing OR orthosis) AND (pain OR functionality OR quality of life).

### 2.2 Data extraction and synthesis

Each full-text document underwent a thorough evaluation for eligibility, conducted independently by two investigators. Data extraction was performed using a pre-designed data extraction form. The extracted data included: year of publication, authors, country, population characteristics, type of spinal orthosis used, modality and duration of orthosis use (hours per day and total length of use), comparator if applicable, and outcome measures (pain, functionality, and quality of life). The collected data were recorded in an Excel spreadsheet for subsequent analysis. Any discrepancies in data extraction were resolved through discussion between the two investigators, or by consulting a third investigator if necessary. This process was carried out manually, without the use of automated tools.

The study findings were integrated using a narrative method, which was necessitated by the heterogeneity in both methodology (including study design and outcomes) and clinical aspects (such as participant and intervention characteristics).

### 2.3 Quality assessment and risk of bias

The risk of bias for included studies was assessed using the Cochrane Risk of Bias 2 (RoB 2) tool for randomized trials and the Risk Of Bias In Non-Randomized Studies of Interventions (ROBINS-I) tool for non-randomized studies. Two independent investigators conducted the evaluations, and any disagreements were resolved through discussion or by consulting a third investigator. Each domain of the RoB 2 and ROBINS-I tools was assessed, including random sequence generation, allocation concealment, blinding of participants and personnel, blinding of outcome assessment, incomplete outcome data, selective outcome reporting, and other potential sources of bias.

### 2.4 Studies characteristics and interventions

A total of 473 records were identified through database searches. After removing duplicates, 267 studies underwent eligibility screening based on titles and abstracts, resulting in the exclusion of 255 records. Twelve studies proceeded to full-text review, but one report was not retrieved, and another was excluded due to its observational design. Thus, ten studies met the inclusion criteria for this mini-review: de Sire et al. (13), Hettchen et al. (14), Kato et al. (15), Kim et al. (16), Li et al. (17), Meccariello et al. (18), Murata et al. (19), Pfeifer et al. (20, 21), and Valentin et al. (22).

The studies included in this review were published between 2004 and 2024 and were conducted in various countries, including Japan (*n* = 3), Germany (*n* = 3), Italy (*n* = 2), Korea (*n* = 1), and Denmark (*n* = 1). The study designs varied, encompassing prospective studies (*n* = 3), randomized controlled trials (RCTs) (*n* = 3), prospective comparative studies (*n* = 1), prospective cohort studies (*n* = 2), and experimental follow-ups (*n* = 1).

Sample sizes ranged from 13 to 284 participants, with a total of 970 participants (68%–100% female), and the mean age was 72 years. Time since osteoporotic vertebral fracture ranged from less than 3 days to more than 6 months, with one study including patients up to 17 years post-fracture. Overall, fracture levels spanned from T1 to L5.

The studies included in the present mini-review reported high heterogeneity in the type of intervention and comparator offered. The most frequent intervention proposed was dynamic orthosis which figured in 6 studies (Spinomed® in 5 studies and Spinfast® in 1 study), then rigid thoracolumbosacral orthosis (TLSO) in 3 studies, and soft lumbar orthosis in 2 studies.

Daily orthosis use varied from 15 min to 24 h per day, with total treatment durations ranging from 3 weeks to 12 months (mean: 13.5 weeks).

Detailed studies' characteristics and intervention data can be found in Table 1.

**Table 1:** Studies’ characteristics and interventions.

Article (Author, Year)	Study design	Sample Size	Sex (%), female	Mean age years	Time since fracture	Intervention	Number of patients	Comparator	Number of patients	Program length (months)
Hettchen et al., 2022 (14)	RCT, semi-blinded	80	80 (100%)	74	>3 months	Spinomed® active 2 h/day for the first 14 days then up to 4–6 h/day	40	no brace	40	4
Kato et al., 2019 (15)	Prospective Randomized	284	-	76	1.5 weeks	rigid TLSO	141	soft TLSO	143	3
Kim et al., 2014 (16)	Prospective Randomized	60	41 (68%)	70	<3 days	1. rigid TLSO 24 h/day	1–20	no brace	20	3
						2. soft back brace 24 h/day	2–20			
Li et al., 2014 (17)	Pilot RCT	55	55 (100%)	82	2–3 weeks	rigid TLSO for 1 week followed by Spinomed® orthosis 3 h/day during rehabilitation and soft lumbar orthosis for the rest of the time	27	rigid TLSO for 1 week followed by soft lumbar orthosis	24	0.75 (3 weeks)
Meccariello et al., 2016 (18)	Prospective comparative	140	100 (71%)	82	1 week	Spinomed®	68	standard 3-point corset	72	6
Murata et al., 2012 (19)	Prospective cohort	55	39 (71%)	75	<1 week	plastic TLSO	53	n/a	n/a	6
Pfeifer et al., 2004 (20)	RCT	62	62 (100%)	72	-	Spinomed® 2 h per day	31	no brace	31	12
Pfeifer et al., 2011 (21)	Crossover RCT	108	108 (100%)	72	<6 months	1. Spinomed® 2 h per day	2–36	no brace for 6 months, then Spinomed	36	12
						2. Spinomed® active 2 h per day	1–36			
de Sire et al., 2024 (13)	Prospective cohort	66	54 (82%)	-	<1 months	Spinfast®	66	n/a	n/a	3
Valentin et al., 2014 (22)	Prospective (experimental follow-up)	13	13 (100%)	71	>3 months	Spinomed® 15 min/day the first 14 days, then up to 2–4 h per day	13	n/a	n/a	3

### 2.5 Main findings

The key findings from the current review are detailed in Table 2. Below, the findings for each type of orthosis are presented:

1. Rigid Orthoses: Rigid orthoses were particularly effective in stabilizing vertebral fractures and preventing spinal deformities during early recovery. Murata et al. found that TLSO braces promoted effective fracture healing, with 88.7% of fractures settled within 6 months, and enabled early mobilization (19). Similarly, Kato et al. demonstrated superior spinal deformity prevention by rigid braces at 12 weeks, although their effectiveness in reducing pain and improving functionality was comparable to soft braces at 48 weeks (15). Despite their stabilizing properties, the long-term benefits of rigid orthoses in terms of pain relief or quality of life were limited.
2. Soft Orthoses: Soft orthoses showed notable efficacy in reducing pain and enhancing mobility in patients with mild to moderate conditions. Li et al. reported significant pain reduction and improved mobility with soft lumbar orthoses, achieving comparable outcomes to rigid braces (17). Kim et al. observed that no-brace, soft-brace, and rigid-brace groups showed similar improvements in pain and functional scores over time, suggesting equivalent long-term recovery benefits (16). The lightweight design of soft orthoses promotes better compliance, making them a favorable choice for extended use.
3. Dynamic Orthoses: Dynamic orthoses, including Spinomed® and Spinfast®, consistently outperformed other types across multiple outcomes, particularly for long-term rehabilitation. Meccariello et al. showed that dynamic orthoses provided greater pain reduction, improved functionality, and fewer complications compared to three-point orthoses (18). Pfeifer et al. documented that Spinomed® improved back extensor strength (up to 73%), posture, and quality of life while reducing pain by 38% and enhancing daily living functionality (20, 21). Valentin et al. reported that Spinomed® III increased back extensor strength by 50%, reduced back pain by 33%, and showed borderline significant improvements in physical functioning (22). Hettchen et al. found that Spinomed active® reduced back pain by 37%, improved kyphosis angle, and enhanced trunk strength and functional performance (e.g., improved chair-rise test results) (14). Similarly, De Sire et al. highlighted that Spinfast® significantly improved pain intensity, physical functioning, and quality of life within 3 months (13).

**Table 2:** Studies’ outcomes and conclusions.

Article (Author, Year)	Outcomes	Conclusions	Risk of bias
Rigid orthosis			
Kato et al., 2019 (15)	Anterior Vertebral Body Compression Percentage (AVBCP) score, visual analog scale (VAS), European Quality of Life-5 Dimensions (EQ-5D-3l), Japanese Orthopaedic Association Back Pain Evaluation Questionnaire (JOABPEQ).	No significant difference in radiological outcomes between rigid and soft braces at 48 weeks for acute vertebral compression fractures, although rigid braces showed better prevention of spinal deformity at 12 weeks. Secondary outcomes, including back pain, quality of life (EQ-5D-3l), and JOABPEQ scores, were also similar between the groups at 48 weeks.	High risk of bias
Murata et al., 2012 (19)	JOABPEQ, local kyphotic angle, fracture settling status, bone mineral density (BMD), fracture status.	Significant improvements were observed in all components of the JOABPEQ at both 3 and 6 months. At 6 months, 88.7% of patients showed fracture settling.	High risk of bias
Soft orthosis			
Kim et al., 2014 (16)	Oswestry Disability Index (ODI), VAS, Short Form-36 (SF-36), anterior body compression ratio.	At 12 weeks post-compression fracture, the ODI score in the no-brace group was not inferior to the soft-brace or rigid-brace groups. During follow-up, overall ODI scores, VAS pain scores, SF-36 scores, and anterior body compression ratios showed no significant group differences, but all improved significantly over time (*p* < 0.001).	High risk of bias
Li et al., 2015 (17)	Pain by a “10-point scale” system, Functional Independence Measure-motor Scores (FIM-motor Scores), Elderly Mobility Scale (EMS), and Modified Functional Ambulation Category (MFAC), thoracic kyphosis angle.	Each group showed significant improvements in pain, mobility, and activities of daily living, with no significant difference observed between the groups.	Some concerns
Dynamic orthosis			
Hettchen et al., 2022 (14)	Back pain intensity by numerical rating scale (NRS), kyphosis angle, trunk strength, functional ability (chair-rise test), back pain-related disability, overall function and disability (Late Life Function and Disability Index), respiratory parameters Forced Vital Capacity (FVC), Forced Expiratory Volume in 1 s (FEV1).	The “Spinomed active” orthosis significantly reduced back pain, improved kyphosis angle and trunk strength, and had positive effects on disability and general function. However, no relevant changes were observed in respiratory parameters. Conclusion: recommended for hyperkyphotic women with osteoporotic vertebral fractures.	Low risk of bias
Meccariello et al., 2016 (18)	Visual Analogue Scale (VAS), Oswestry Low Back Pain Disability Questionnaire (OLBPDQ). morphological evaluation, FEV1.	There was no difference in vertebral deformity or kyphosis between the groups at any time point. However, Group 2 experienced significantly less pain and disability at 3 and 6 months. Complications occurred in 14 out of 23 participants (60.8%) in the 3-point group and 3 out of 20 participants (15%) in the Spinomed group.	Some concerns
Pfeifer et al., 2004 (20)	height, kyphosis, extensor strength, body sway, FEV1, average pain by Miltner's rating scale, LDL disability, LDL self-care, well being.	The orthosis group showed significant improvements compared to the control group in back extensor strength (73%), pain reduction (38%), abdominal strength (58%), height, kyphosis angle (11%), body sway (25%), well-being (15%), and daily living (27%). Compliance was high, and no complications were reported.	High risk of bias
Pfeifer et al., 2011 (21)	height, kyphosis, extensor strength, body sway, FEV1, average pain by Miltner's rating scale, LDL disability, LDL self-care, well being.	Both orthosis groups showed significant improvements compared to the control group in back extensor strength, abdominal strength, pain, balance, kyphosis angle, well-being, and daily living. There was no significant difference between the two orthosis groups, and compliance was high in both groups 1 and 2.	Some concerns
de Sire et al., 2024 (13)	VAS, SPPB, EQ-VAS.	Improvement in pain intensity, physical functioning, and QoL.	High risk of bias
Valentin et al., 2014 (22)	Back extensor strength (maximal isometric muscle strength), back pain by 11-point numerical ranking scale, physical functioning (SF-36 health survey).	Increased back extensor strength by 50% (*p* = 0.01), reduced back pain by 33%, and improved physical functioning by 6.5 points.	High risk of bias

### 2.6 Risk of bias assessment and confounder management

The quality of the included studies was assessed using the Cochrane RoB2 tool for randomized trials and the ROBINS-I tool for non-randomized studies, overall judgments are reported in Table 2. Randomized trials showed varying levels of bias, with some at low risk and others at higher risk, particularly in deviations from intended interventions. As a result, these studies had some concerns regarding bias, and conclusions should be interpreted with caution. For non-randomized studies, risks ranged from low to serious, particularly due to confounding.

Overall, while the studies provide valuable insights, their limited quality requires cautious interpretation and further research is needed to confirm the findings.

The included studies, moreover, varied significantly in their approaches to monitoring confounders, particularly the use of analgesics. Some studies, such as Hettchen et al. (14) and Kato et al. (15), systematically tracked analgesic use over time, either through regular follow-up assessments or questionnaires, and reported no significant differences between groups. Others, including Pfeifer et al. (20, 21) and Valentin et al. (22), provided baseline data on analgesic consumption but did not monitor changes during the study period, limiting the ability to interpret their impact on outcomes. De Sire et al. (13) reported detailed data on pain medications, highlighting variability among participants but acknowledged the lack of systematic screening for other potential confounders. Conversely, studies such as Kim et al. (16), Li et al. (17), Meccariello et al. (18), and Murata et al. (19) did not address the use of analgesics or confounders related to pain management, presenting a gap in methodological rigor. These inconsistencies underscore the need for standardized protocols to account for confounding factors in future research.

## 3 Discussion

This mini-review revealed that while spinal orthoses, including rigid, soft, and dynamic types, generally improved pain, functional outcomes, and quality of life in patients with osteoporotic vertebral fractures, no single orthosis consistently outperformed others across all measures.

More in detail, our investigation revealed several key insights into the efficacy of different spinal orthoses for managing pain, improving functionality, and enhancing quality of life in patients with osteoporotic vertebral fractures. Across the studies, no significant differences were observed between rigid and soft braces in terms of pain relief, functional improvement, or quality of life, although rigid braces were more effective in preventing spinal deformity in the short term, 12 weeks. However, long-term radiological outcomes at 48 weeks were similar between the two types of braces (15). Moreover, Murata et al. supports the role of rigid orthoses, such as the plastic TLSO, in providing critical biomechanical support and facilitating early mobilization, though it is noted that such benefits may not always translate into significantly better long-term outcomes compared to softer or dynamic options (19). Interestingly, the absence of a brace did not result in inferior clinical outcomes when compared to either soft or rigid braces, with all groups showing comparable improvements in pain and functionality over time (16). This indicates that while rigid braces may offer short-term benefits in preventing deformity and providing biomechanical support, their overall impact on pain relief and functional improvement is comparable to that of soft braces. Thus, the choice between rigid and soft braces may depend more on patient-specific factors and preferences rather than on significant differences in clinical effectiveness. The systematic review published by Newman et al. in 2016 reported that rigid orthoses were associated with higher complication rates compared to softer alternatives (6). This suggests that while rigid braces might offer certain benefits, they may come with increased risks and complications. Soft braces, in contrast, often present fewer complications and may provide a comparable level of clinical benefit without the added risk, making them a potentially safer choice for managing these stable fractures.

On the other hand, studies on dynamic braces, such as those by Hettchen et al. (14), Pfeifer et al. (20, 21), Valentin et al. (22), and De Sire et al. (13), present a more favorable picture for dynamic orthoses. Li et al. (17) and Meccariello et al. (18) investigated the general efficacy of dynamic orthoses and found that they contribute to significant improvements in pain reduction and functional mobility, with Meccariello et al. indicating superior outcomes in pain and disability reduction compared to three-point orthoses, and Li et al. showing that Spinomed® and soft lumbar orthoses demonstrated similar efficacy in pain reduction and functional mobility gains during the subacute stage (17). Pfeifer's et al. studies on the Spinomed® dynamic brace demonstrated notable improvements in back extensor strength, kyphosis angle, and quality of life, with minimal adverse effects, supporting its use as an effective non-pharmacological treatment (20, 21). Similarly, the Spinomed® III orthosis was effective in enhancing back extensor strength and reducing pain, with borderline significant improvements in physical functioning (22). Moreover, Hettchen et al. stated that the Spinomed® active orthosis significantly reduced back pain intensity and kyphosis angle while improving trunk strength and disability outcomes in hyperkyphotic women with vertebral fractures older than 3 months. Although respiratory function did not improve, the findings highlight orthosis as an effective option for addressing chronic back pain and related physical limitations in this population (14). However, as noted by Pieroh et al., the positive results associated with Spinomed® have been challenged by concerns about study quality and potential biases, indicating that while Spinomed shows potential, further high-quality research is necessary to confirm its effectiveness and address issues related to reproducibility (12). Finally, Spinfast® dynamic spinal orthosis, a more recent development, has demonstrated improvements in pain management, physical functioning, and quality of life in older patients with vertebral fragility fractures, according to De Sire et al. (13).

These findings collectively suggest that while different spinal orthoses offer varying degrees of benefit, dynamic braces such as the Spinomed® and Spinfast® offer promising non-pharmacologic options for managing osteoporotic vertebral fractures. This orthosis appears to address some limitations of previous models and provides promising results. Nonetheless, both Spinomed® and Spinfast® should be evaluated with careful consideration of their respective evidence bases. The existing studies suggest benefits from dynamic braces, but additional well-designed research is needed to establish their definitive role and to ensure that these benefits are consistently reproducible across diverse patient populations.

Our nuanced results are in line with previous reviews. Pieroh et al. noted a preference for soft or dynamic orthoses over rigid braces. Even though, despite some studies showing potential benefits, the highest-quality research indicated that patients treated without an orthosis had comparable outcomes to those using one. This highlights the limited and mixed evidence supporting the routine use of spinal orthoses (12). In contrast, Kweh et al. provided moderate-quality evidence supporting spinal orthoses for improving vertebral stability and functional outcomes in elderly patients (23). Similarly, Goodwin et al. highlighted the inconsistent evidence for orthotic devices and taping, noting that while Spinomed® showed some benefits, the overall quality of studies was limited (10).

In addition, Newman et al. conducted a systematic review whose key findings showed some promising results for subacute and longer-term rehabilitation. Specifically, semirigid thoracolumbar orthoses (TLO) and weighted kypho-orthoses (WKO) demonstrated potential benefits in improving strength, pain, posture, and balance (6). Rzewuska et al. et al. found low to moderate evidence supporting spinal orthoses for medium-term pain relief and disability reduction, which resonates with our findings on dynamic braces. However, this review also pointed out the overall low-quality evidence for conservative treatments, including orthoses, and the need for better-designed studies (9).

Lastly, Sanchez-Pinto-Pinto et al. demonstrated that dynamic hyperextension braces significantly reduced thoracic kyphosis and improved back extensor strength and quality of life, aligning with our results on the effectiveness of dynamic braces in improving these outcomes (24).

Overall, the findings suggest that while dynamic orthoses like Spinomed® and Spinfast® show promise in improving patients' outcomes, the comparative effectiveness of different orthotic types remains a subject of ongoing research. Moreover, the above-mentioned reviews collectively call for larger, more rigorous studies to better determine the efficacy of orthoses and to explore their role within comprehensive rehabilitation programs. The current evidence suggests that while spinal orthoses may offer some benefits, especially in specific contexts like subacute rehabilitation, their overall effectiveness and the risk of complications warrant cautious interpretation and further investigation.

This mini-review has several limitations to consider. The included studies exhibited significant heterogeneity in study design, participant characteristics, types of orthoses, and outcome measures. Methodological quality varied, with some studies lacking blinding or randomization, potentially introducing bias. Inconsistent follow-up periods limited the assessment of long-term efficacy and safety. Additionally, the management of confounders, particularly related to pain medications, varied considerably across studies. While some studies systematically monitored and reported the use of analgesics and other pharmacological interventions, others provided only baseline data or no information at all. This inconsistency limits the ability to fully account for the potential impact of pain medications on the reported outcomes. There is also a risk of publication bias, as studies with negative results may be underrepresented, and the review's restriction to English-language studies could lead to language bias. These factors highlight the need for more rigorous randomized controlled trials with standardized measures and longer follow-up periods to better evaluate the efficacy of spinal orthoses for osteoporotic vertebral fractures.

In conclusion, the choice of spinal orthosis should be tailored to individual patient needs, considering fracture characteristics and the specific advantages and limitations of each orthotic type. While spinal orthoses can help manage pain and improve function in osteoporotic vertebral fractures (OVFs), the variability in study designs and outcomes prevents definitive conclusions. Soft orthoses appear non-inferior to rigid braces, which may carry higher complication risks, while dynamic hyperextension orthoses like Spinomed® and Spinfast® show promise in enhancing trunk muscle strength and functionality. However, further high-quality randomized trials are needed to solidify these findings and guide clinical practice.
